# The Magnitude of the Association between Human Papillomavirus and Oral Lichen Planus: A Meta-Analysis

**DOI:** 10.1371/journal.pone.0161339

**Published:** 2016-08-29

**Authors:** Junxian Ma, Jinshan Zhang, Yan Zhang, Tingting Lv, Jie Liu

**Affiliations:** 1 Department of Rheumatology and Immunology, Tangdu hospital, The Fourth Military Medical University, Xi'an, China; 2 Department of Human Anatomy and Histology and Embryology, The Fourth Military Medical University, Xi'an, China; Penn State University School of Medicine, UNITED STATES

## Abstract

**Background:**

The role of human papilloma virus (HPV) in oral lichen planus (OLP) is controversial.

**Objectives:**

The primary aim of the current study is to calculate the pooled risk estimates of HPV infection in OLP when compared with healthy controls.

**Methods:**

Bibliographic searches were conducted in three electronic databases. Articles on the association between HPV and OLP were selected from case-control studies or cross-sectional studies, following predefined criteria. Pooled data were analyzed by calculating odds ratios (OR) and 95% confidence interval (CI).

**Results:**

Of the 233 publications identified, 22 case-control studies met the inclusion criteria. Collectively, 835 cases and 734 controls were available for analysis. The summary estimate showed that OLP patients have significantly higher HPV prevalence (OR: 6.83; 95% CI: 4.15–11.27) than healthy controls. In subgroup analyses, the association of HPV and OLP varied significantly by geographic populations. The ORs ranged from 2.43 to 132.04. The correlation of HPV and erosive-atrophic oral lichen planus (EA-OLP) (OR: 9.34) was comparable and well above that of HPV and non-EA-OLP (OR: 4.32). Among HPV genotypes, HPV 16 showed an extremely strong association with OLP (OR: 11.27), and HPV 18 showed a relatively strong one (OR: 6.54).

**Conclusion:**

In conclusion, a significant association was found between HPV and OLP. The strength of the association varied across geographic populations, clinical types of OLP, and HPV genotypes. The results suggest that HPV might play an important causal role in OLP and in its malignant to progression.

## Introduction

Oral lichen planus (OLP) is a common chronic autoimmune disorder, which may present epithelial thickening or atrophy with or without ulceration [1]. Clinically, there are six different types: papule, reticular, plaque, atrophic, erosive and bullous. The most common type is reticular. All types of OLP can be pooled in 2 clinical groups: erosive-atrophic forms (EA-OLP), including erosive, atrophic, bullous and mixed EA variants; and non-erosive-atrophic forms (non-EA-OLP), involved papule, reticular, plaque and mixed non-EA variants. EA-OLP is more prone to malignant transformation than non-EA-OLP [2]. The prevalence of HPV in OLP has been reported to range from 0.5 to 2.2%, varying according to geographic location [3].

Although the etiology of OLP is still unknown, it is generally accepted to be a T-cell-mediated inflammatory disease [1]. The reaction of these specific CD8+ T cells is similar to what occurs during a viral infection, in which a virus can act as a cytoplasmic antigen or induce the expression of host cell proteins, resulting in a differing host cell protein profile [4]. In this way, exploring the possibility of viral involvement in the pathogenesis of OLP is improving.

As early as 1987, the association between HPV and OLP was reported [5]. In one study published prior to 1998, 107 OLP samples were studied, and 23% were HPV-positive. The first three most prevalent HPV types were HPV 6, 11, and 16. Then 1929 normal oral mucosa samples were tested for HPV DNA, and 11% were found to be positive [6]. The rate of HPV in OLP was twice as high as in normal cases. On the basis of these studies, the case-control studies of HPV and OLP correlation were increasingly growing.

However, results have been conflicting: some studies seemed to show that HPV did play an important role in OLP [7–10], but others disagreed [11–13]. There have been some reviews of the HPV and OLP, but most of them are qualitative analyses. Very few reviews have included quantitative analysis. There was one meta-analysis of HPV in oral carcinoma and oral potentially malignant disorders (OPMD). OLP was a sub-analysis of OMPD, and the OR of the link between HPV and OLP was 5.12 [14]. The results indicated a strong association, but the articles included were limited, a more comprehensive meta-analysis is needed.

Two English databases (PubMed and Web of Science) and one Chinese database (CNKI) were screened in order to collect more articles. Finally, 22 case-control studies were included to perform this systematic review and meta-analysis. The primary aim of the current paper was to clarify the magnitude of the association between HPV and OLP, and to get a further understanding of it concerning with different geographical populations, different OLP clinical types and HPV genotypes.

## Materials and Methods

### Data sources

A systematic search was performed in PubMed (www.ncbi.nlm.nih.gov/pubmed), Web of Science (SCI) (http://webofknowledge.com/) and CNKI (http://epub.cnki.net/) to screen relevant literature, until the last search update on September 21, 2015. The search terms were (lichen planus or oral lichen planus or LP or OLP) and (human papillomavirus or HPV). The reference lists of selected papers were searched to identify additional studies.

### Data extraction

**Fig 1:**
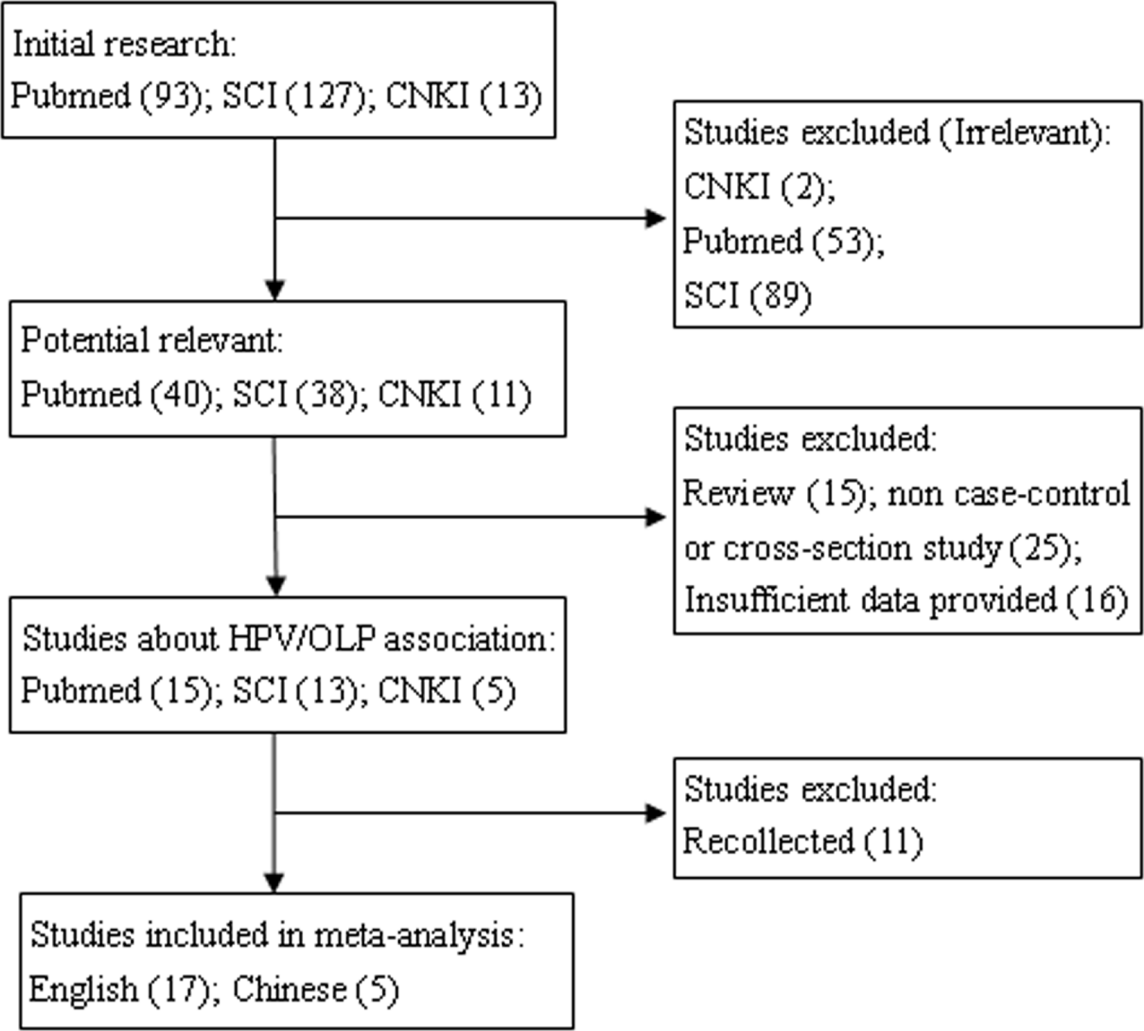
Flow chart of literature searches for this meta-analysis.

The screening process was carried out by two investigators (J. Ma and J. Zhang) independently under the same criteria (Fig 1). Disagreements about eligibility were settled by consensus with a third investigator (Y. Zhang). The following details were recorded from each study: first author, publication date, country, clinical type of OLP, number of OLP patients and healthy controls, test methods, and HPV genotypes (Table 1).

**Table 1:** Characteristics of the 22 included studies in this meta-analysis.

Reference	Nation	Clinical type	OLP(n/N)	Control(n/N)	Detection method	HPV genotypes
Pol CA *et al*., 2015	India		21/30	0/30	IHC	HPV16
Arirachakaran P *et al*., 2013	Thailand		1/37	0/37	PCR	NA
Yildirim B *et al*., 2011	Turkey		14/65	0/15	IHC	HPV16
Debanth S *et al*., 2009	India		6/6	3/35	PCR	HPV16, 18, 31, 33, 35, 39, 45, 51,52, 56, 58, 59,68
Fehér E *et al*., 2009	Hungary		39/119	3/72	PCR	HPV6.11.16.18.31.33
Razavi SM *et al*., 2009	Iran		9/29	1/14	PCR	HPV18
Szarka K *et al*., 2009	Hungary		39/119	3/72	PCR	NA
		EA-OLP	26/61	3/72		
		non-EA-OLP	13/58	3/72		
Yu Hong *et al*., 2007	China		33/57	3/20	PCR	HPV16
Cianfriglia F *et al*., 2006	Italy		3/15	2/10	ISH	NA
Giovannelli L *et al*., 2006	Italy		12/49	11/49	PCR	NA
Campisi G *et al*., 2004	Italy		14/71	5/90	PCR	HPV16.18.31.6
		EA-OLP	9/44	5/90		
		non-EA-OLP	5/27	5/90		
Ma Jian *et al*., 2003	China		26/30	5/18	PCR	NA
OFlatharta C *et al*., 2003	Ireland		10/38	0/20	PCR	HPV16
Giovannelli L *et al*., 2002	Italy		9/34	5/90	PCR	NA
Li Hui *et al*., 2000	China		9/30	3/40	PCR	NA
Sand L *et al*., 2000	Sweden		6/22	0/12	NA	NA
Qi Yanchun *et al*., 1999	China		9/30	3/40	PCR	NA
Lei Lei *et al*., 1997	China		9/22	2/10	PCR	NA
		EA-OLP	3/4	2/10		
		non-EA-OLP	6/18	2/10		
Vespe M *et al*., 1997	Germany		3/7	0/33	PCR	HPV16.18.31
Boyd AS *et al*., 1996	USA		11/13	0/10	PCR	NA
Cox M *et al*., 1993	UK		3/4	3/5	PCR	HPV16
Maitland NJ *et al*., 1987	UK		7/8	5/12	PCR	HPV16

n: numbers of HPV positive subjects; N: numbers of total subjects; IHC: immunohistochemical staining; PCR: polymerase chain reaction; ISH: in situ hybridization; NA: not available; EA-OLP: erosive-atrophic oral lichen planus; non-EA-OLP: non-erosive-atrophic oral lichen planus.

### Selection criteria

Studies included in the meta-analysis had to meet the following criteria: (i) address the relationship between HPV and OLP; (ii) full-text papers; and (iii) original case-control or cross-sectional studies. Studies were excluded if they included any of the following: (i) reviews; (ii) incomplete data; and (iii) republished articles or reused data.

### Quality assessment

Results of observational studies are greatly influenced by the design of the research. To guarantee the quality of the meta-analysis, each included paper was carefully assessed using the standard proposed by Chalmers *et al*. [15], including selection of subjects, comparability between groups and outcome presented. The selected studies were scored on an ordinal star scale from 1 to 9, with higher scores representing higher quality.

### Statistical analysis

The meta-analysis was conducted using RevMan5, a copyrighted freeware developed by the Cochrane Collaboration, for preparing and maintaining reviews (http://www.cochrane-net.org/revman). Heterogeneity among different studies was measured by calculating *χ*^*2*^ and *I*^*2*^, if *P* < 0.05 and *I*^*2*^ > 50% were considered statistically significant. The primary outcome was reported as pooled odds ratios (OR) with 95% confidence intervals (CI), calculated using the random-effects model when heterogeneity existed or the fixed-effects model when no heterogeneity was found. To investigate potential for publication bias, the funnel plot asymmetry of the included studies was evaluated. Subgroup analysis was undertaken for geographic differences, clinical types of OLP, and HPV genotypes.

### Sensitivity analysis

Low-quality studies (five stars or fewer) were excluded to investigate potential selection bias. Articles with different methods of HPV detection were analyzed to assess detection bias.

## Results

### Overall information on the studies included

The process of search strategy is showed in Fig 1. From the 233 articles identified through an initial research, 144 were excluded for lack of relevance to OLP. After further reading, 15 papers were excluded for reviews, 25 papers were excluded without case-control or cross-sectional design, and 16 studies with insufficient data were excluded. Eleven papers were both collected in PubMed and SCI database. In the end, 22 articles met the criteria. The main information of the 22 selected studies is listed in Table 1. The analysis covered 12 countries, 5 of Asia, specifically China [16–20], India [9, 10], Iran [21], Thailand [22], and Turkey [23]; 6 in Europe, specifically Italy [12, 13, 24, 25], Hungary [26, 27], England [5, 11], Germany [8], Ireland [28], and Sweden [29]; 1 in North American, specifically the United States [7].

### Results of quality assessment

On the basis of the criteria described previously [15], 20 studies were evaluated as superior quality, with scores of six or more stars, and 2 studies were of low quality, with five stars or fewer (Table 2). Nine articles lacked a definition or diagnosis of OLP [5, 7, 11, 12, 16–20]. Three papers did not clearly explain the selection criteria for the controls [8, 11, 25], and 1 paper used diseased patients as controls [7]. Seventeen papers either did not give information about the sex of subjects or the sexes between cases and controls were incomparable [5, 7, 8, 10–13, 17–21, 23, 24, 26, 28, 29].

**Table 2:** Assessment of quality and score for 22 studies included.

Reference	Selection of subjects				Comparability				Outcome	Score
	(1)	(2)	(3)	Age		Sex	Living area	Race		
Pol CA *et al*., 2015	*	*	*	*			*	*	**	8
Arirachakaran P *et al*., 2013	*	*	*	*		*	*	*	**	9
Yildirim B *et al*., 2011	*	*	*				*	*	**	7
Debanth S *et al*., 2009	*	*	*	*		*	*	*	**	9
Fehér E *et al*., 2009		*	*	*			*	*	**	7
Razavi SM *et al*., 2009	*	*	*				*	*	**	7
Szarka K *et al*., 2009	*	*	*	*		*	*	*	**	9
Yu Hong *et al*., 2007		*	*	*		*	*	*	**	8
Cianfriglia F *et al*., 2006		*	*				*	*	**	6
Giovannelli L *et al*., 2006	*	*	*				*	*	**	7
Campisi G *et al*., 2004	*	*	*				*	*	**	7
Ma Jian *et al*., 2003		*	*				*	*	**	6
OFlatharta C *et al*., 2003	*	*	*				*	*	**	7
Giovannelli L *et al*., 2002	*	*				*	*	*	**	7
Li Hui *et al*., 2000		*	*				*	*	**	6
Sand L *et al*., 2000	*	*	*				*	*	**	7
Qi Yanchun *et al*., 1999		*	*				*	*	**	6
Lei Lei *et al*., 1997	*	*	*				*	*	**	7
Vespe M *et al*., 1997	*	*					*	*	**	6
Boyd AS *et al*., 1996		*					*	*	**	5
Cox M *et al*., 1993		*					*	*	**	5
Maitland NJ *et al*., 1987		*	*				*	*	**	6

(1) Was there a specific definition of the diagnosis of this disease in the article?

(2) Were the selection criteria for the patients in the study specifically described?

(3) How representative was the control group with respect to the source population of cases enrolled? Comparability: Were the groups comparable with respect to age, sex, living area or race? The “Outcome” item was scored for double asterisks (**) in cells; others were scored for a single asterisk (*).

### Meta-analysis

**Fig 2:**
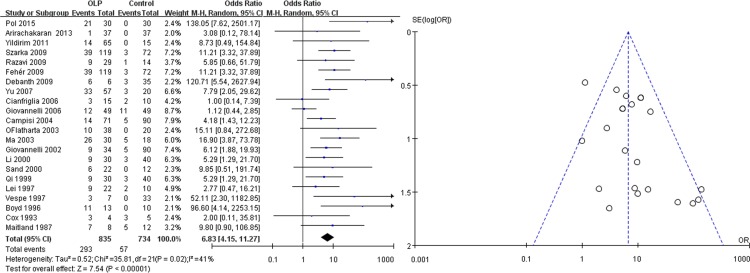
Forest and funnel plots of 22 included studies on the association between HPV and OLP.

As shown in Fig 2, the studies had a total of 835 patients and 734 controls, of which 293 cases (35.09%) and 57 controls (7.77%) were HPV positive. The *χ2* and *I*^*2*^ were 35.81 (*P* = 0.02) and 41%, respectively, suggesting heterogeneity. The random-effects model was used to analyze the data. The pooled OR was 6.83 (95%CI: 4.15–11.27), and the overall effect *Z* value was 7.54 (*P* < 0.00001), which indicated a strong association between HPV and OLP. The visual examination of the symmetry of the funnel plot did not suggest a large publication bias (Fig 2).

### Subgroup analyses

We further conducted subgroup analyses of all included studies based on geographic population, OLP clinical types and HPV genotypes respectively, to determine the influencing factors that may impact the overall results.

#### Association of HPV with OLP in terms of geographic population

The 22 articles included 12 countries in Asia, Europe, and North America. The association varied significantly by geographic population (Table 3). In Asia (OR: 9.37), it was strongest in India (OR: 132.04), followed by Turkey (OR: 8.73), China (OR: 6.58), Iran (OR: 5.85), and Thailand (OR: 3.08). There were 11 studies that reported the association of OLP and HPV (OR: 5.15) in Europe, including Germany (OR: 52.11), Ireland (OR: 15.11), Hungary (OR: 11.21), Sweden (OR: 9.85), England (OR: 5.34) and Italian (OR: 2.43). The relationship between OLP and HPV was especially significant in the U.S. study (OR: 96.60).

**Table 3:** Association of HPV with OLP in terms of geographic population.

Region	Geographical	Articles	OLP	Control	OR (95% CI)
	population	involved	(n/N)	(n/N)	
Asia	Asia	10	137/336	20/259	9.37[5.38,16.30]
	India	2	27/36	3/65	132.04[14.92,1168.20]
	Turkey	1	14/65	0/15	8.73[0.49,154.84]
	China	5	86/169	16/128	6.58[3.44,12.60]
	Iran	1	9/29	1/14	5.85[0.66,51.79]
	Thailand	1	1/37	0/37	3.08[0.12,78.14]
Europe	Europe	11	145/486	37/465	5.15[2.56,10.34]
	Germany	1	3/7	0/33	52.11[2.30, 1182.85]
	Ireland	1	9/29	1/14	15.11[0.84,272.68]
	Hungary	2	78/238	6/144	11.21[3.32,37.89]
	Sweden	1	6/22	0/12	9.85[0.51,191.74]
	England	2	10/12	8/17	5.34[0.91,31.45]
	Italy	4	38/169	23/239	2.43[1.38,4.27]
North American	America	1	11/13	0/10	96.60[4.14,2252.15]

n, positive number of subjects; N, total number of subjects

#### Association of HPV with OLP in terms of clinical type

**Fig 3:**
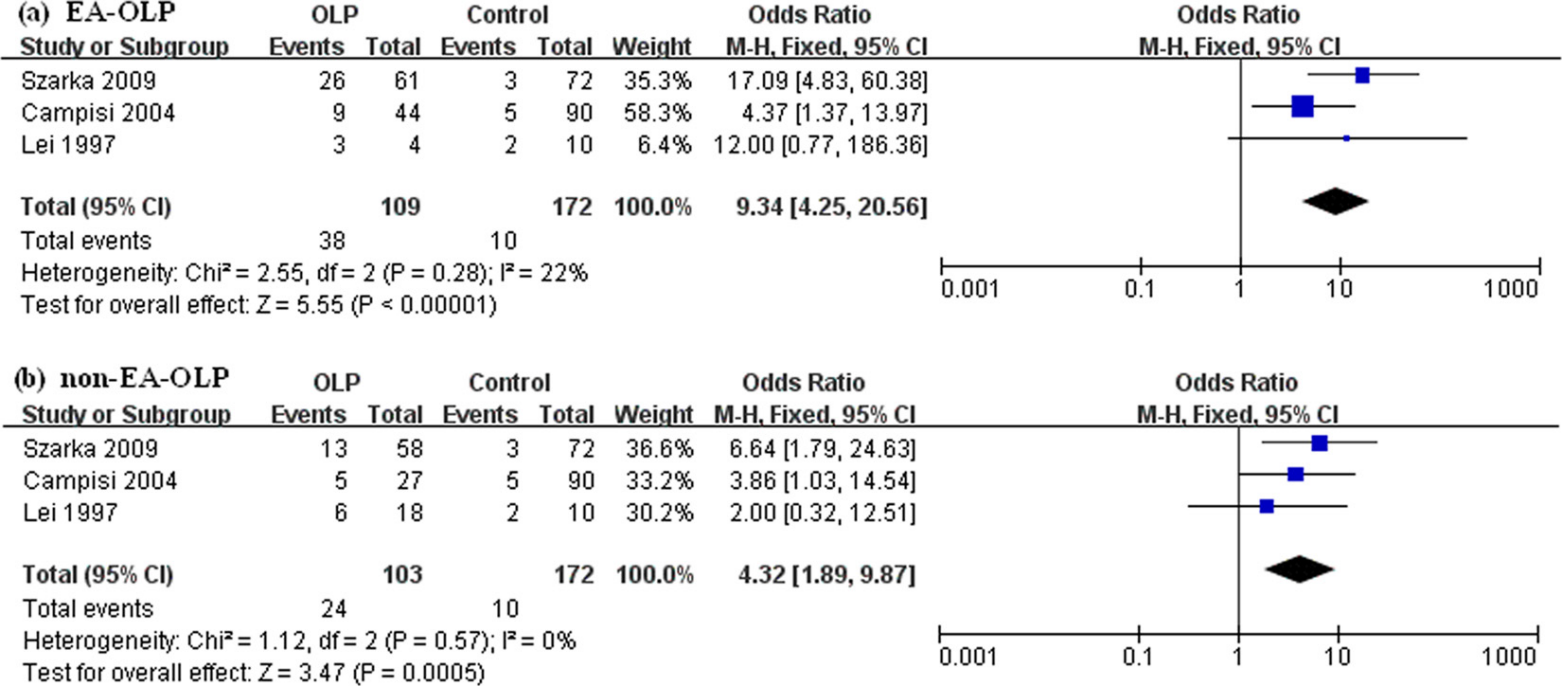
Forest plots of the association between HPV and OLP in terms of clinical type. (a) EA-OLP and (b) non-EA-OLP.

Two types of OLP were involved, EA-OLP and non-EA-OLP. There were only 3 studies mentioned [20, 24, 27], including Hungary (OR: 17.09 vs 6.64), Italy (OR: 4.37 vs 3.86), and China (OR: 12.00 vs 2.00). As shown in Fig 3, the prevalence of HPV differed significantly between the more risky EA-OLP (OR: 9.34; 95%CI: 4.25–20.56) and non-EA-OLP (OR: 4.32; 95%CI: 1.89–9.87).

#### Association of HPV with OLP in terms of HPV genotype

**Fig 4:**
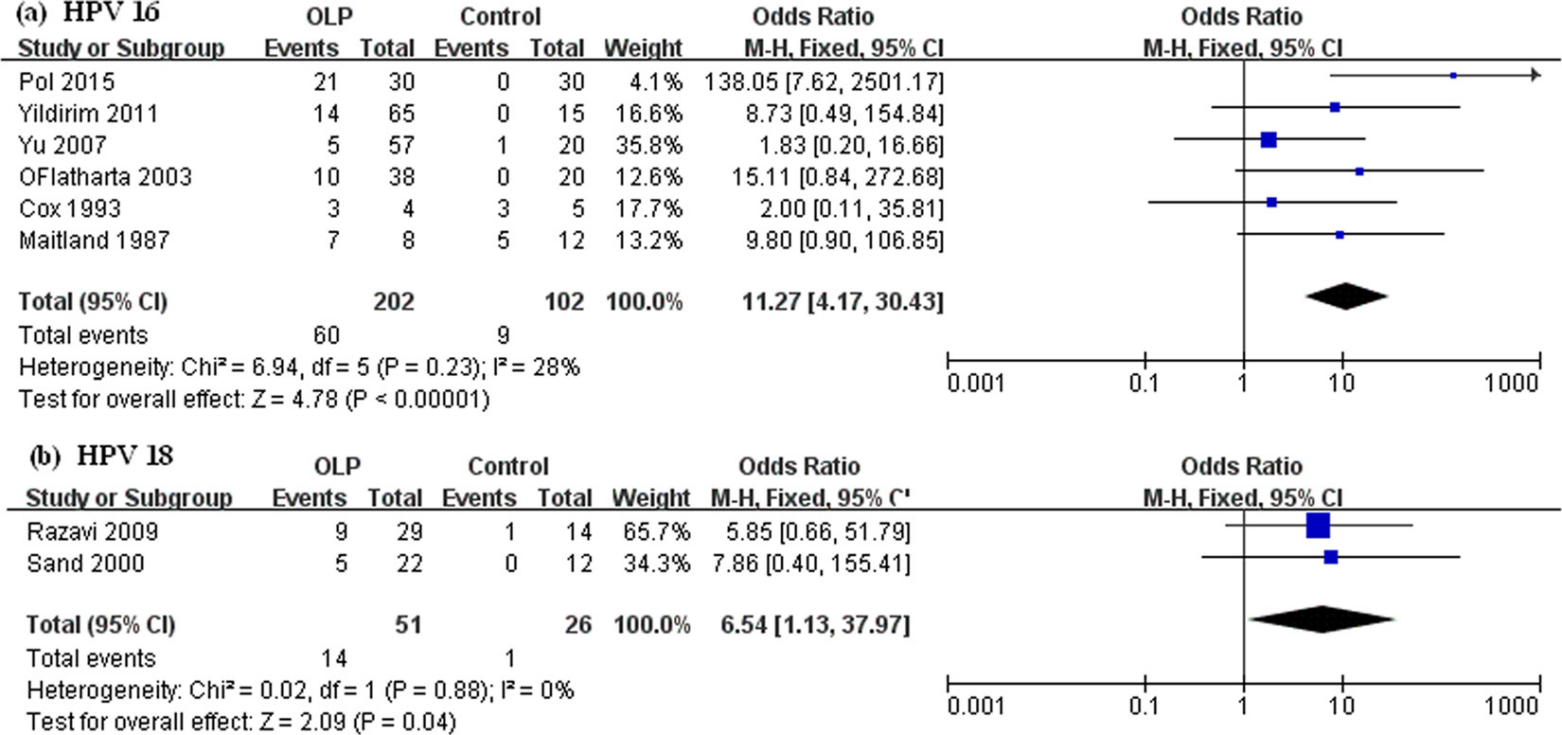
Forest plots of the association between HPV and OLP in terms of HPV genotypes. (a) HPV16 and (b) HPV18.

The association of OLP and HPV varied across different HPV genotypes. Among the 22 included articles, HPV16 was the most frequently reported genotype, and HPV18 was the second. ORs of the correlation between HPV16 and OLP from 1.83 to 138.05, the pooled OR of 6 papers was 11.27 (95%CI: 4.17–30.43) (Fig 4A). Only Razavi SM *et al*. [21] and Sand L *et al*. [29] reported the association between HPV18 and OLP (pooled OR: 6.54) (Fig 4B).

### Sensitivity analysis

**Fig 5:**
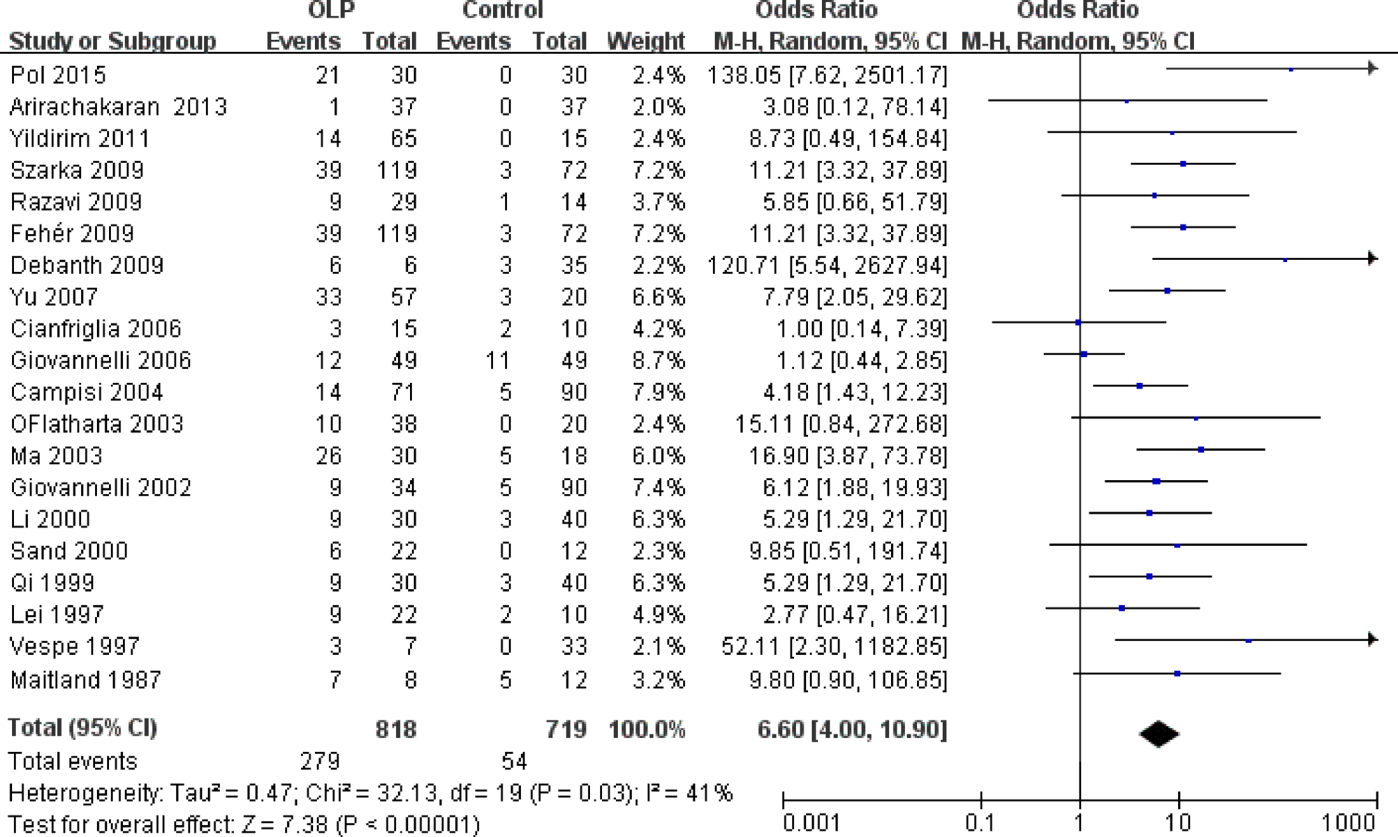
Forest plots of the association between HPV and OLP in high-quality studies.

The exclusion of low-quality studies did not change the summary estimate significantly, OR was 6.60 (Fig 5). Comparing results across detection methods was not considered feasible, given that only 2 studies used IHC, 1 used ISH, and 19 used PCR.

## Discussion

OLP is a chronic inflammatory mucocutaneous disease whose pathogenesis is still the object of much speculation. Different mechanisms by which OLP may develop have been hypothesized in recent years. The association between OLP and viral infections is one of the most controversial positions. The most widely studied viruses in OLP are HPV and hepatitis-C (HCV) [30].

HPVs are small, double-stranded, and circular DNA viruses. There are approximately 40 genotypes known to infect the oral cavity and urogenital tract [31]. The incidence of HPV oral infection has increased in recent decades, and the infection rate seems to be associated with age and gender [32]. Given the U.S. Annual Report on cancer in 2013, the incidence of HPV-positive oropharyngeal (OP) cancers has increased proportionally, while the total incidence of cancer has recently declined [33]. The latest analysis of U.S. cancer registry data showed that the number of HPV-positive OP cancers diagnosed each year may exceed that of invasive cervical cancers by 2020 [32]. In this way, the association of HPV with OLP and oral cancer has received more attention.

This meta-analysis showed a significantly strong association between OLP and HPV. The pooled OR showed that OLP patients have about a 7-fold higher risk of HPV infection than controls, which was consistent with results reported by Syrjänen *et al*. in 2011 [14]. Besides, we found that the correlation of HPV and OLP vary not only according to geographic populations, but also clinical types of OLP and HPV genotypes. This is the most comprehensive meta-analysis of this issue ever performed.

The current study showed there to be wide variations in the association of OLP and HPV with regard to different geographic populations, which was consistent with results reported by Lodi G *et al*. [34]. Among the 12 countries included, the ORs of HPV/OLP association ranged from 1.00 to 138.05. Studies performed in Italy (OR: 1.12, 1.00) and the U.K. (OR: 2.00) did not show any significant relationship between HPV and OLP [11–13]. In contrast, patients with OLP in India (OR: 132.04), the United States (OR: 96.60), Germany (OR: 52.11), Ireland (OR: 15.11), and Hungary (OR: 11.21) showed an extremely strong association. In this way, efforts into exploring the correlation between HPV and OLP should focus mainly on epidemiological studies of different populations.

The most common clinical forms of OLP are EA-OLP and non-EA-OLP. In the current meta-analysis, the association between HPV and EA-OLP (OR: 9.34) was comparable and stronger than the association of HPV and non-EA-OLP (OR: 4.32). The risk of progression to malignancy for non-EA-OLPs was approximately 0.5%, but in EA-OLPs it was at least 3.5–4.0% during similar follow-up periods [27]. All this adds up to a hypothesis that differences in HPV prevalence may influence OLP malignant potential. This could shed light on the possibility of a potential prognostic application of HPV detection in OPMD.

Here, the association strength between HPV 16 and OLP was extremely high (OR: 11.27), and HPV types16 has been classified as high-risk (HR) type and reported to be associated with malignancy [35]. Besides, HPV16 has also been reported to be the most common infectious HPV genotype in oral squamous cell carcinoma (OSCC). Its prevalence was 16%. OLP is an OPMD of the oral mucosa with a transformation rate of 0–6.25% [2, 34], so whether HPV 16 has a causal role in OLP malignancy, a long-term follow up is needed to determine.

Recent data from case-control and meta-analytic studies suggested that HPV is a causal factor of the development of several types oropharyngeal and oral cavity squamous carcinomas [36–39]. However, only small proportion of individuals who become infected with HPV will develop OSCC. A recent study showed that patient individual susceptibility to HPV infection, and other biomarkers may be related to OSCC development [40]. Other studies showed that the prognosis, age of onset, and incidence for men and women were different between HPV-positive and HPV-negative oral cancer patients [33]. In this way, determining the HPV infection status of HPV-associated OPMD and OSCC patients may be important to prognosis, treatment, and prevention strategies.

The major limitations of the current meta-analysis are the detection/diagnostic bias and selection bias of the case-control studies that were included. The 22 articles evaluated in this meta-analysis used three kinds of HPV detection methods: PCR, ISH and IHC. PCR is exquisite sensitive and widespread used, it is generally considered as the gold standard in most meta-analysis [14]. However, PCR detection is prone to false positive results because of contamination may occur during sampling, processing and PCR protocols [7]. ISH can be more sensitive in cases in which only a few cells in the sample tissue contain high copy numbers of the virus [41], but its results can influenced by the quality of the sample (e.g. frozen or fixed) [42]. In this way, detection and selection biases limit the usefulness of these results due to possible errors of these kinds. In future work, a uniform research standard should be established to perform a more precise comparison of the results.

## Conclusion

In conclusion, this systematic review and meta-analysis showed a significant association between HPV and OLP. All sub-analyses strongly and consistently indicated this association. Because of the lack of prospective cohort studies, it was not possible to take a position on the relationship between HPV infection and oral malignancy. More prospective cohort studies are needed to formally confirm the role of HPV as an etiological agent of OLP, and a uniform research standard should be established to produce get more convincing results.

## Supporting Information

S1 PRISMA Checklist PRISMA 2009 Checklist. (DOC)
